# Association of Hospital Practices and Early Postnatal Support with Breastfeeding Outcomes in Premature and Term Infants

**DOI:** 10.3390/children13050642

**Published:** 2026-05-03

**Authors:** Andreea Teodora Constantin, Ioana Roșca, Leonard Năstase, Alexandru Dinulescu, Alina Turenschi, Gabriel-Petre Gorecki, Ciprian Andrei Coroleuca, Elena Poenaru, Daniela Eugenia Popescu

**Affiliations:** 1 Department of Pediatrics and Department of Neonatology, National Institute for Mother and Child Health “Alessandrescu-Rusescu”, 20382 Bucharest, Romania; 2 Faculty of Midwifery and Nursery, University of Medicine and Pharmacy “Carol Davila”, 020021 Bucharest, Romania; 3 Clinical Hospital of Obstetrics and Gynecology “Prof. Dr. P. Sirbu”, 060251 Bucharest, Romania; 4 Neonatology Department, National Institute for Mother and Child Health “Alessandrescu-Rusescu”, 011061 Bucharest, Romania; 5 Faculty of Medicine, University of Medicine and Pharmacy “Carol Davila”, 020021 Bucharest, Romania; 6 Emergency Clinical Hospital for Children “Grigore Alexandrescu”, 011743 Bucharest, Romania; 7 Pediatrics Department, Pediatric Hospital, 100097 Ploiești, Romania; 8 Faculty of Medicine, Titu Maiorescu University, 031593 Bucharest, Romania; 9 Department of Anesthesia and Intensive Care, CF 2 Clinical Hospital, 011464 Bucharest, Romania; 10 Department of Obstetrics-Gynecology and Neonatology, “Victor Babeș” University of Medicine and Pharmacy, 300041 Timișoara, Romania

**Keywords:** breastfeeding, premature and term infants, motherhood, postnatal support

## Abstract

**Highlights:**

**What are the main findings?**

- Exclusive breastfeeding rates remain suboptimal (49.3%), with delayed mother–infant contact, low rates of early initiation, and inconsistent hospital practices across settings.
- Preterm birth and lack of rooming-in are strongly associated with delayed breastfeeding initiation and significantly lower rates of exclusive breastfeeding.

**What are the implications of the main findings?**

- Improving early postnatal practices (immediate contact, rooming-in, and timely initiation) represents a key, modifiable opportunity to increase exclusive breastfeeding rates.
- Standardized, evidence-based breastfeeding support—particularly for preterm infants—should be prioritized across all maternity settings to reduce disparities and improve outcomes.

**Abstract:**

Background/Objectives: Exclusive breastfeeding offers optimal benefits for infant nutrition and health and increases maternal involvement, bonding and interactions. This study aimed to explore breastfeeding practices among mothers in Romania and identify risk factors associated with low exclusive breastfeeding rates. Methods: A cross-sectional online survey was conducted between September and December 2025, targeting mothers in Romania via social media platforms. The questionnaire, developed specifically for this study, collected data on sociodemographic characteristics, birth and neonatology variables, hospital practices, feeding intentions, community influences, and breastfeeding outcomes. Responses were analyzed using Fisher’s exact tests and multivariable logistic regression. Results: A total of 357 complete questionnaires were analyzed. Cesarean section was the most frequent mode of delivery (54.6%), while immediate mother–infant contact after birth was reported by only 35.6% of mothers, and breastfeeding initiation within the first hour occurred in 10.6% of cases. Overall, 49.3% of mothers reported exclusive breastfeeding, 35.3% mixed feeding, and 15.4% exclusive formula feeding. Women who delivered in private hospitals reported earlier mother–infant contact, more frequent encouragement to initiate breastfeeding, and earlier breastfeeding initiation compared with those delivering in public hospitals. Preterm birth was associated with delayed breastfeeding initiation, reduced rooming-in, and lower rates of exclusive breastfeeding up to six months. In multivariable logistic regression, rooming-in was independently associated with higher odds of exclusive breastfeeding (aOR = 2.798, 95% CI: 1.779–4.401), while lack of lactation support was associated with lower odds (aOR = 0.546, 95% CI: 0.302–0.987). No significant associations were observed for timing of initial maternal–infant contact (aOR = 1.084, 95% CI: 0.679–1.733) or encouragement from medical staff to initiate breastfeeding (aOR = 1.207, 95% CI: 0.721–2.020). Conclusions: Our study highlights current breastfeeding practices and associated hospital factors in Romania. However, significant challenges remain in supporting and encouraging mothers to optimally feed their infants. Additional investment and bold policy action are needed to promote and support breastfeeding from the first hour of life, for both term and preterm infants, in all maternity hospitals in Romania.

## 1. Introduction

Breastfeeding is beneficial for mothers, children and society, being widely recognized by leading health organizations around the world as the normative standard for virtually all infants due to its health benefits for infants and their mothers. The World Health Organization (WHO) and the American Academy of Pediatrics (AAP) generally recommend exclusive breastfeeding for approximately the first six months and continued breastfeeding, along with the introduction of solid foods, for at least two years after birth, as long as this is mutually desired by the parent and infant [1,2,3]. Breastfeeding provides nutrients specific to the human species and supports the normal development of infants by providing non-nutritional growth factors, immune factors, hormones and other bioactive components that can act as biological signals; it can decrease the incidence and severity of infectious diseases, enhance neurological development, decrease the incidence of obesity and other chronic diseases in childhood, and decrease the incidence and severity of atopic diseases. Breastfeeding also benefits the mother’s health by increasing maternal metabolism, has contraceptive effects in the case of exclusive breastfeeding, and is associated with a decrease in the occurrence of postmenopausal breast cancer and osteoporosis. It confers benefits within the community by reducing health costs and provides benefits related to the expenditure on infant formula [4,5,6,7,8]. These recommendations are strongly supported by numerous other medical and professional organizations, including the American Academy of Family Physicians (AAFP), based on both short- and long-term benefits for mother and child [9].

Hospital policies should include strategies for promoting breastfeeding, including placing mother and baby in the same room during hospitalization and ensuring skin-to-skin contact immediately after birth [5].

The first 1000 days of life represent a critical period of rapid growth and development, during which lifelong health trajectories can be shaped. Nutrition in the neonatal period is very important, being the foundation of life. During this period, nutrition plays an important role, and adverse exposures can induce long-lasting structural, functional, and epigenetic adaptations that increase susceptibility to chronic diseases in adulthood [10,11,12,13,14]. Early-life preventive strategies in pediatrics extend beyond nutrition and also include early identification of inherited conditions that may influence long-term health outcomes, as recently demonstrated in Romanian children with familial hypercholesterolemia [15,16,17].

Human milk is particularly important for preterm infants, providing protection against necrotizing enterocolitis, late-onset sepsis, and other complications of prematurity [18,19,20,21,22]. Unfortunately, despite the strong recommendations, actual breastfeeding practice falls short of these goals in many countries and cultures. In the United States, more than 80 percent of birthing parents initiate breastfeeding, but less than 60 percent continue through six months postpartum, and less than 40 percent are breastfeeding at 12 months [23]. Similar trends are observed in other settings, including Romania, where exclusive breastfeeding rates remain suboptimal despite high maternal intention to breastfeed. In a recent cross-sectional survey, although most mothers intended to breastfeed, only a little over half achieved exclusive breastfeeding, with factors such as cesarean delivery, lack of rooming-in, and pacifier use negatively influencing outcomes [24,25].

As such, promoting and supporting breastfeeding is not merely a personal choice but a collective responsibility with profound implications for the health and prosperity of future generations.

Despite the extensive literature on the benefits of breastfeeding, there remains a significant gap in understanding how specific hospital-related practices and early postnatal support mechanisms interact to influence outcomes within the Romanian healthcare system, particularly when comparing preterm and term infant cohorts. The primary objective of this study was to evaluate the association between hospital practices—including mode of delivery, rooming-in, and professional lactation support—and exclusive breastfeeding rates at discharge. We hypothesized that: (1) infants delivered in private maternity settings would have higher exclusive breastfeeding rates due to increased access to prenatal education and early support, and (2) preterm infants would experience significantly lower rates of “Golden Hour” initiation and exclusive breastfeeding compared to term infants due to systemic clinical barriers and mother–infant separation.

## 2. Materials and Methods

We conducted a cross-sectional observational study based on responses to an online survey on breastfeeding among Romanian mothers. The questionnaire, consisting of 46 questions, was distributed online on platforms such as Facebook and Instagram and to different groups on WhatsApp (Meta Platforms, Inc., Menlo Park, CA, USA; version current between September and December 2025). Online communities for mothers were targeted in the urban and rural regions of Romania.

A self-administered online questionnaire was developed specifically for this study and distributed between September and December 2025. Appendix A and Appendix B provide the Romanian version and a version translated in English of the questions included in our questionnaire. The survey was conducted in Romanian and included both multiple-choice and single-response questions. Completion time was approximately 5 min. The questionnaire was anonymous, and no identifying data were collected, in accordance with GDPR regulations. It addressed the following thematic domains:

- Sociodemographic data: Maternal age, education level, family income, area of residence (urban/rural), smoking status, and number of children.
- Perinatal factors: Maternal age at first birth, birth method (vaginal or cesarean), gestational age, birth weight, and timing of first skin-to-skin contact.
- Hospital feeding practices and support: Rooming-in status, type of feeding in hospital, advice and support received from medical staff regarding breastfeeding initiation and technique, and use of pacifiers.
- Breastfeeding behavior and intentions: Whether the mother planned to breastfeed, initiation and duration of breastfeeding, timing of milk production onset, and whether difficulties were encountered (e.g., latch problems, pain, mastitis, perceived low supply).
- Use of formula: Whether the infant received formula and at what point, and whether there was any external pressure (from family, medical staff, or community) to introduce formula.
- Attitudes and perceptions: Maternal experience with breastfeeding (rated from very positive to very difficult), perceived facilitators of successful breastfeeding (e.g., partner support and lactation consultants), and sources of information (e.g., prenatal classes, online forums, and medical professionals).

Participants were instructed to respond with reference to their youngest child.

The questionnaire was inspired by existing surveys on breastfeeding practices and attitudes, adapted to reflect the Romanian cultural and healthcare context. Face validity was evaluated through pre-testing with five mothers with recent breastfeeding experience. Their feedback on clarity, content, and length was used to refine the final version of the survey.

Exclusive breastfeeding (EBF) was defined according to World Health Organization (WHO) standards as the infant receiving only breast milk (including expressed milk or milk from a wet nurse), with no other liquids or solids—not even water—with the exception of oral rehydration salts, drops, or syrups consisting of vitamins, minerals, or medicines.

The study was approved by the Ethics Committee of the Clinical Hospital of Obstetrics and Gynecology “Prof. Dr. Panait Sîrbu”, Bucharest, Romania, under approval number 5828/8 April 2025, and was conducted respecting the Helsinki Declaration for Human Rights.

Statistical analysis was performed using IBM SPSS Statistics, version 22.0 (IBM Corp., Armonk, NY, USA). Categorical variables are presented as numbers and percentages. Comparisons between women who gave birth in public hospitals and those who gave birth in private hospitals were conducted using Pearson’s chi-square test when all observed cell frequencies were ≥10. When at least one observed cell frequency was <10, Fisher’s exact test (two-sided) was applied.

Variables included in the multivariable logistic regression model were selected based on clinical relevance and statistical significance in univariate analysis (*p* < 0.05). A stepwise backward elimination approach was applied to identify independent predictors of exclusive breastfeeding. The multivariable model included key hospital-related and perinatal variables considered potential confounders of exclusive breastfeeding, such as mode of delivery, rooming-in, timing of first mother–infant contact, breastfeeding encouragement by medical staff, and lactation support during hospitalization.

To identify factors associated with exclusive breastfeeding, logistic regression analyses were conducted. First, univariate logistic regression analyses were performed to evaluate the association between each independent variable and the outcome variable (exclusive breastfeeding). Crude odds ratios (ORs) with 95% confidence intervals (CIs) were calculated.

Variables considered clinically relevant or showing an association in univariate analysis were subsequently included in a multivariate logistic regression model to identify independent predictors of exclusive breastfeeding. Adjusted odds ratios (aORs) with 95% confidence intervals were reported.

Model fit was assessed using the Hosmer–Lemeshow goodness-of-fit test, with a *p*-value > 0.05 indicating an adequate fit. Additional model performance indicators, including Nagelkerke R^2^ and −2 log likelihood, were also reported.

A total of 30 independent statistical tests were performed, corresponding to the main questionnaire variables. To account for multiple comparisons and control the family-wise error rate, the Bonferroni correction was applied, resulting in an adjusted significance threshold of *p* < 0.00167 (0.05/30). Inferential conclusions were based on Bonferroni-adjusted *p*-values, while unadjusted *p*-values (*p* < 0.05) are reported for descriptive and exploratory purposes. All tests were two-tailed.

## 3. Results

The database includes 357 complete questionnaires. All descriptive analysis includes the full sample of respondents.

For inferential statistical analyses examining hospital-related practices, participants who reported home birth were excluded, as hospital-related variables were not applicable to this group.

The study cohort’s mean chronological age at the time of the survey was 36.4 years ± 8.15. Regarding reproductive history, the mean age at primiparity (first childbirth) was 28.5 years ± 5.2.

Table 1 provides an overview of our study population and participants’ characteristics.

### 3.1. Sociodemographic Characteristics of the Study Population

Most respondents lived in urban areas (77%), while 23% resided in rural settings. There were no statistically significant differences between the two groups (mothers who gave birth in a public vs. private hospital).

Regarding educational level, the majority had higher education, with 42.6% holding a university degree and 38.1% having post-university studies, while a smaller proportion reported post-secondary or vocational education.

Household net monthly income varied, with 24.9% reporting incomes above 12,000 RON (approx. 2355 EUR), followed by 27.7% between 8001 and 12,000 RON, 36.7% between 4000 and 8000 RON, and 10.6% below 4000 RON.

Most participants were non-smokers (72.5%), while 21.3% reported active smoking and a small proportion reported occasional smoking.

### 3.2. Obstetric and Perinatal Characteristics

More than half of the participants (54.6%) gave birth by cesarean section without general anesthesia, while 38.7% delivered vaginally and a smaller proportion by cesarean section under general anesthesia. Among women who delivered by cesarean section, the most frequent indication was planned cesarean section for medical reasons (50.7%), followed by (27.1%) previous cesarean delivery and emergency cesarean section (22.2%).

Most infants (79.6%) were born at term (>37 weeks of gestation). Birth weight was between 2500 and 4000 g in 81.8% of cases, while 5.6% weighed more than 4000 g at birth.

The majority of mothers reported delayed first contact with their newborn, with 35.6% holding their infant immediately after birth, while 37.3% first held their child after several hours. The most commonly reported reasons for delayed contact were maternal anesthesia (38.1%), neonatal medical needs (32.6%), and unknown reasons (25.7%).

### 3.3. Early Breastfeeding Practices During Hospitalization

Most participants gave birth in a public maternity hospital (73.9%), while 23.8% delivered in a private hospital; a very small proportion reported a home birth.

Almost seventy percent (69.7%) of mothers reported being encouraged by medical staff to initiate breastfeeding during the first contact with their infant. Initiation of breastfeeding occurred immediately after birth in 10.6% of cases, while 31.1% initiated breastfeeding more than 12 h postpartum and 15.4% after more than 24 h. A small proportion reported never breastfeeding.

During maternity hospitalization, 52.4% of infants stayed with their mothers in a rooming-in system, while 47.6% were cared for in neonatal units. Regarding feeding methods during hospitalization, 52.4% received both breast milk and formula, 17.4% received infant formula, and 25.2% were breastfed.

**Figure 1:**
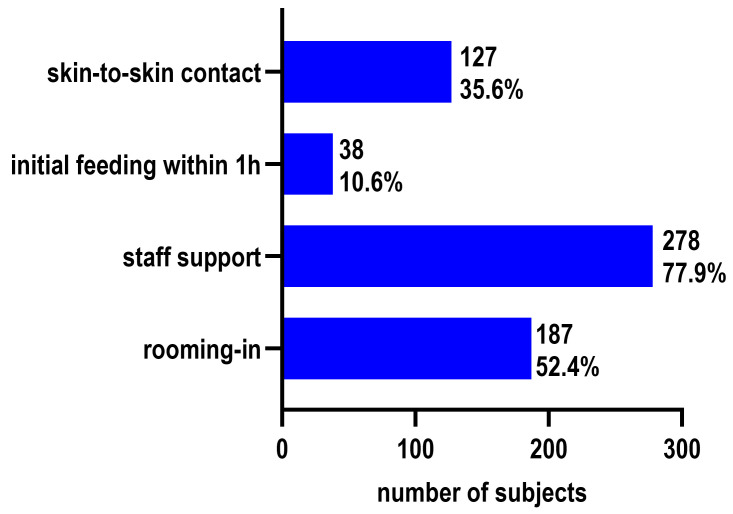
Frequency of early clinical practices and hospital support (*n* = 357). Bars indicate the number and percentage of mothers experiencing skin-to-skin contact, breastfeeding initiation within the first hour, rooming-in, and receiving professional encouragement.

During hospitalization, 77.9% of mothers reported receiving breastfeeding support from medical staff, while 19.9% reported receiving no assistance (Figure 1).

### 3.4. Infant Feeding Practices and Breastfeeding Outcomes

**Figure 2:**
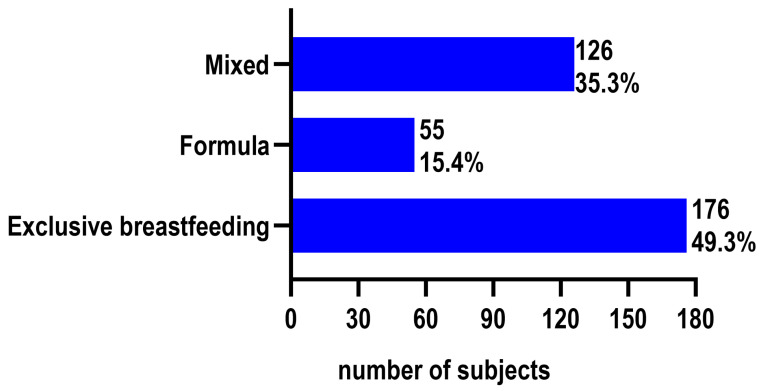
Prevalence of infant feeding methods among the total study population (*n* = 357). Categories include exclusive breastfeeding, mixed feeding, and formula feeding.

Overall, 49.3% of mothers reported exclusive breastfeeding, 35.3% practiced mixed feeding, and 15.4% fed their infants exclusively with formula (Figure 2).

Among respondents who were still within the breastfeeding period (*n* = 302), 70.2% reported breastfeeding, while 29.8% were not breastfeeding at the time of survey completion. Exclusive breastfeeding until six months was achieved by a subset of mothers, while others reported intentions to continue exclusive breastfeeding in infants younger than six months.

Pacifier use was common, with 25.8% offering a pacifier from birth, 32.2% introducing it after the first six weeks, and 42% reporting no pacifier use.

### 3.5. Breastfeeding Support, Barriers, and External Influences

More than a quarter (25.8%) of mothers sought help from a certified lactation consultant, while 12.3% reported that they would have liked to but found the services too expensive.

A significant proportion of mothers (35%) reported being discouraged by family members or acquaintances regarding breastfeeding, whereas 9.2% reported experiencing pressure from healthcare professionals to discontinue breastfeeding.

Maternal medical conditions potentially affecting lactation were reported by 12% of participants, most commonly thyroid disorders and polycystic ovary syndrome. Additionally, 15.7% of mothers reported using medication during the breastfeeding period.

Breastfeeding difficulties related to infant conditions were reported by 11.8% of participants, including prematurity, ankyloglossia, reflux, and neonatal complications.

### 3.6. Sources of Breastfeeding Information

The most frequently reported sources of breastfeeding information were family and friends (50.1%), online forums and social media groups (37.8%), and medical professionals, including family physicians (43.7%) and lactation consultants (26.1%). Formal prenatal breastfeeding education was reported by 23.2% of participants.

### 3.7. Maternal Characteristics and Postnatal Care Practices by Type of Maternity Unit

After excluding mothers that gave birth at home, we analyzed 349 cases. Out of them, 264 (75.6%) gave birth in public hospitals and 85 (24.4%) in private maternity units.

Table 2 provides an overview of our study population and participants’ characteristics in public hospitals vs. private practice.

There were no statistically significant differences between the two groups in terms of living environment (rural vs. urban; *p* = 0.271). Education was almost significant between the two groups (*p* = 0.051), with most mothers with university degrees choosing to give birth in private hospitals, although the difference did not reach statistical significance.

On the other hand, family revenue was significantly different between the two groups (*p* = 0.001), with women who gave birth in private hospitals more frequently reporting incomes > 12,000 RON/month, while incomes < 4000 RON/month were more common in the public hospital group.

The number of children was significantly different between groups (*p* = 0.030), with women in private hospitals more frequently having 1–2 children, while those in public hospitals more often had ≥3 children. No significant differences were observed regarding smoking (*p* = 0.671).

The type of delivery differed significantly between the two groups (*p* = 0.001). Cesarean section, particularly without general anesthesia, was significantly more frequent in private hospitals, whereas vaginal birth was more common in public hospitals. The reasons for cesarean section also differed significantly (*p* = 0.004), with planned cesarean sections reported more frequently in private hospitals.

Gestational age at birth did not differ significantly between groups (*p* = 0.101); however, birth weight showed significant differences (*p* = 0.042), with infants weighing ≥ 2500 g being more frequent in the private hospital group.

The timing of the first time the mother held her baby differed significantly between groups (*p* < 0.001). Women who gave birth in private hospitals more frequently reported immediate or early contact, while delays of several hours were more common in public hospitals.

The duration of the first time holding the baby also differed significantly (*p* < 0.001), with longer durations reported more often in private hospitals. Encouragement from medical staff to put the baby to the breast was significantly more frequently reported in private hospitals (*p* = 0.013).

Timing of breastfeeding initiation also showed significant differences (*p* < 0.001), with women in private hospitals more frequently reporting breastfeeding within the first hour after birth.

Participation in prenatal breastfeeding classes was significantly more frequent among women who gave birth in private hospitals (*p* = 0.001). Although receiving breastfeeding assistance in the maternity ward was more frequent in private hospitals, the difference did not reach statistical significance (*p* = 0.059).

No significant differences were observed regarding rooming-in, type of feeding in the maternity ward, or pacifier use.

No significant differences were identified between groups regarding breastfeeding planning before birth, difficulties encountered, breastfeeding duration, or exclusive breastfeeding up to 6 months. However, seeking the services of a lactation consultant was significantly more frequent among women who gave birth in private hospitals (*p* < 0.001).

### 3.8. Preterm Birth and Breastfeeding Outcomes

Preterm birth was significantly associated with several perinatal characteristics and breastfeeding-related practices.

The mode of delivery differed significantly between groups (*p* < 0.001), with preterm infants being born more frequently by cesarean section without general anesthesia (73.6% vs. 51.3%), while vaginal delivery was more common among term infants (42.6% vs. 16.7%). The main indication for cesarean section also differed significantly (*p* < 0.001), with emergency cesarean sections reported more frequently among preterm births (27.8% vs. 10.5%).

**Figure 3:**
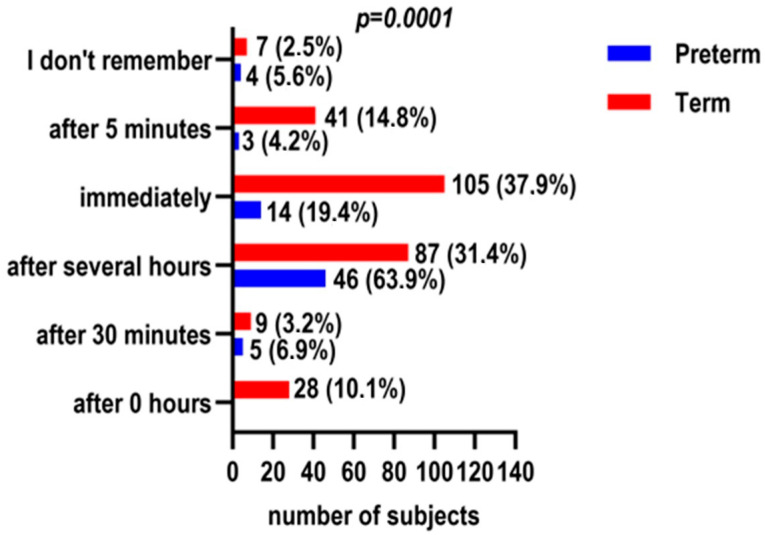
Maternal recall of the timing of initial physical contact (“first holding”) following delivery, stratified by gestational age.

Mother–infant contact also differed significantly. Mothers of preterm infants reported holding their baby for the first time more frequently, several hours after birth (63.9% vs. 31.4%), whereas immediate or early contact was more common among term infants (*p* < 0.001) (Figure 3). When contact was delayed, neonatal medical needs were reported, as expected, more frequently in the preterm group (40.3% vs. 15.2%) (*p* < 0.001). The duration of the first mother–infant contact also differed significantly (*p* = 0.017), with shorter initial holding times reported more often among mothers of preterm infants.

**Figure 4:**
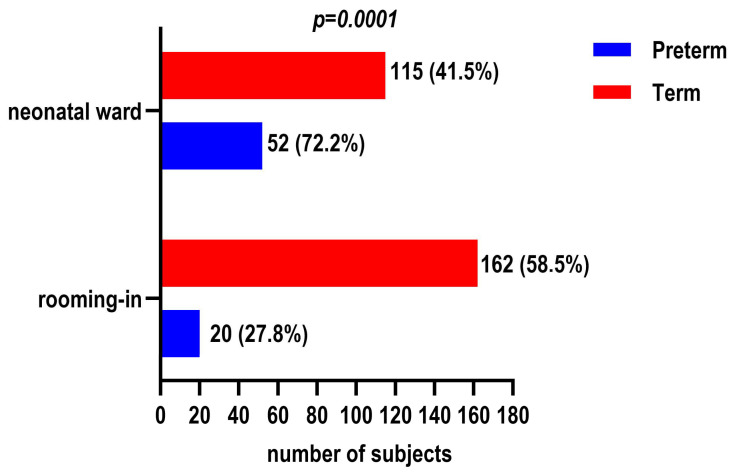
Distribution of hospital placement (rooming-in vs. neonatal ward) according to infant maturity.

Initiation of breastfeeding differed significantly between groups (*p* < 0.001). Mothers of preterm infants more frequently reported initiating breastfeeding more than 24 h after birth (38.9% vs. 9.7%), while earlier initiation was more common among term infants. Rooming-in was significantly less frequent among preterm infants (27.8% vs. 58.5%), who were more often cared for in the neonatal unit (*p* < 0.001) (Figure 4). Feeding practices during hospitalization also differed significantly (*p* < 0.001), with preterm infants more frequently receiving formula or expressed breast milk, while direct breastfeeding was more common among term infants.

**Figure 5:**
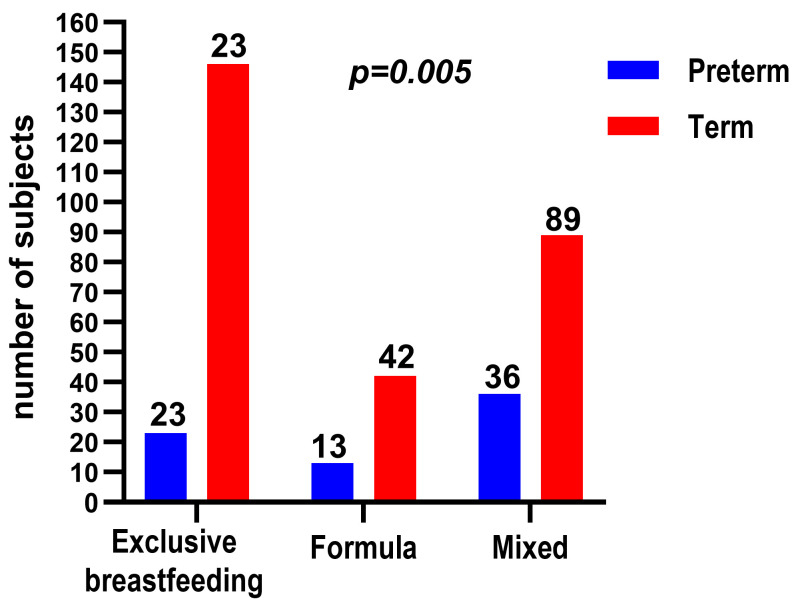
Comparison of exclusive breastfeeding, formula feeding, and mixed feeding rates between preterm (*n* = 72) and full-term (*n* = 285) infants.

Recommendations regarding feeding frequency differed significantly (*p* < 0.001), with mothers of preterm infants more often advised to feed at fixed intervals, whereas feeding on demand was more frequently recommended for term infants. In addition, mothers of preterm infants reported significantly higher rates of perceived pressure from medical staff to discontinue breastfeeding (16.7% vs. 7.2%, *p* = 0.031). Finally, preterm birth was associated with lower rates of exclusive breastfeeding (31.9% vs. 52.7%, *p* = 0.005) and a lower likelihood of exclusive breastfeeding up to six months (34.7% vs. 51.6%, *p* = 0.029) (Figure 5).

A subgroup analysis was conducted to compare breastfeeding outcomes based on the degree of prematurity, using a 34-week gestational age threshold—a key clinical milestone for the development of suck–swallow–breathe coordination (Table 3). Maternal sociodemographic characteristics, including residence, educational level, household income, smoking status, parity, and attendance at prenatal breastfeeding classes, were similar across gestational age groups (all *p* > 0.05). In contrast, several perinatal characteristics differed significantly. Mothers of preterm infants were more likely to deliver by cesarean section, particularly emergency cesarean section, among those delivering before 34 weeks’ gestation (*p* < 0.001). As expected, birth weight increased with gestational age (*p* < 0.001). Earlier gestational age was also associated with delayed first maternal contact, reduced provider-initiated breastfeeding support at initial holding, later initiation of breastfeeding, and a higher likelihood of neonatal admission rather than rooming-in (all *p* < 0.05). These findings highlight the greater perinatal challenges encountered by mothers delivering preterm infants.

### 3.9. Predictors of Exclusive Breastfeeding: Univariate and Multivariate Analysis

Univariate logistic regression analysis identified several factors associated with exclusive breastfeeding (Table 4).

Maintaining the newborn in the same room as the mother (rooming-in) was strongly associated with an increased likelihood of exclusive breastfeeding (OR = 3.09, 95% CI: 1.99–4.78, *p* = 0.0001), as was vaginal delivery (OR = 2.73, 95% CI: 1.74–4.29, *p* < 0.001).

Prematurity was associated with significantly lower odds of exclusive breastfeeding (OR = 0.42, 95% CI: 0.24–0.73, *p* = 0.002).

The timing of the initial maternal–infant contact (“holding the child for the first time”) was not significantly associated with exclusive breastfeeding (OR = 1.26, 95% CI: 0.83–1.92, *p* = 0.277). Similarly, encouragement from medical staff to initiate breastfeeding during the first interaction did not show a statistically significant association (OR = 1.11, 95% CI: 0.70–1.75, *p* = 0.659).

Lactation support during hospitalization showed a borderline association (OR = 0.61, *p* = 0.074), while timing of first holding, encouragement from medical staff, and household income were not significantly associated with exclusive breastfeeding.

In the multivariate analysis, maintaining the newborn in the same room as the mother (rooming-in) remained a significant independent predictor of exclusive breastfeeding (OR = 2.72, 95% CI: 1.71–4.33, *p* < 0.001) (Table 5).

**Figure 6:**
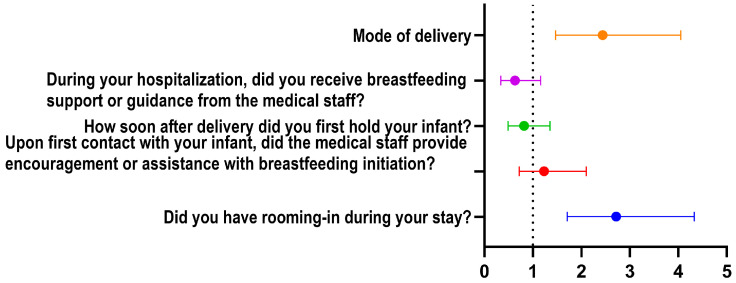
Factors associated with breastfeeding outcomes. The forest plot illustrates the association between clinical hospital practices and exclusive breastfeeding. Results are expressed as odds ratios (OR) with 95% confidence intervals (CI). The vertical dotted line represents the null effect (OR = 1.0). Points to the right of the line indicate a positive association, while points to the left indicate a negative association. Significance is achieved when the 95% CI does not cross the null line.

Similarly, vaginal delivery was independently associated with higher odds of exclusive breastfeeding (OR = 2.44, 95% CI: 1.47–4.05, *p* = 0.001). Prematurity, timing of first holding, encouragement from medical staff, lactation support, and household income were not significantly associated with exclusive breastfeeding after adjustment for confounders (Figure 6).

The model demonstrated good fit, as indicated by the Hosmer–Lemeshow test (χ^2^(8) = 3.913, *p* = 0.865), and explained a modest proportion of variance (Nagelkerke R^2^ = 0.171).

## 4. Discussion

In this cross-sectional study conducted on 357 mothers from Romania, exclusive breastfeeding was reported by less than half (49.3%), indicating suboptimal adherence to international recommendations despite a predominantly urban (77%) and highly educated sample (the majority had higher education (80.7%)). A multivariate analysis identified a high percentage of cesarean delivery (61.3%), almost half of mothers (46.5%) initiating breastfeeding more than 12–24 h postpartum, and the absence of rooming-in (47.6% of infants cared for in neonatal units) as independent risk factors for delayed initiation and continuation of breastfeeding.

In our study, rooming-in was associated with exclusive breastfeeding; mothers who remained in continuous contact with their infants showed nearly threefold higher odds of maintaining exclusive breastfeeding. A 2019 meta-analysis of prospective controlled studies comparing rooming-in with nursery care, which reported full or partial breastfeeding up to six months, found no significant differences in full breastfeeding at 3, 4 or 6 months, except for a small increase in the proportion of infants receiving partial breastfeeding in the rooming-in group at 3–4 months of age [26]. In contrast, a 2022 study conducted in Taiwan reported that full (24 h) rooming-in was significantly associated with continued exclusive breastfeeding at 3 months postpartum [27]. Similarly, Chertok et al. found that the organizational factors of rooming-in and early breastfeeding were significantly associated with exclusive breastfeeding outcomes [28].

A particularly concerning finding in our study is the high prevalence of cesarean sections (54.6% of all deliveries). This rate significantly exceeds the WHO-recommended threshold of 10–15% [29] and was even more pronounced in private maternity units, where it reached 72.9%. Our data confirms that surgical delivery is associated with delayed breastfeeding initiation, with only 10.6% of mothers achieving contact within the “Golden Hour”. This finding is consistent with a recent meta-analysis of 33 countries, which found that C-sections are associated with a 46% lower prevalence of early breastfeeding initiation due to maternal pain and delayed lactogenesis [30]. Furthermore, the disparity between private (72.9%) and public (50.4%) hospitals in our cohort mirrors trends in other middle-income countries, where private-sector C-sections are often significantly higher and more likely to disrupt early skin-to-skin contact [31]. These results suggest that the “C-section epidemic” in Romania is not merely a clinical necessity but a systemic practice that hinders alignment with the Baby-Friendly Hospital Initiative (BFHI) standards, which are proven to improve outcomes regardless of birth mode [32,33].

These associations can be interpreted through the lens of breastfeeding physiology. Early initiation of breastfeeding and uninterrupted mother–infant contact plays a critical role in triggering lactogenesis II through the coordinated release of prolactin and oxytocin. Delays in initiation, maternal–infant separation, and early formula supplementation may disrupt this feedback loop, leading to reduced milk production and lower breastfeeding confidence. In this context, hospital practices do not merely reflect organizational differences but may directly influence biological processes essential for successful breastfeeding establishment [34,35].

According to data reported by UNICEF and the World Health Organization (WHO), the global rate of exclusive breastfeeding for infants under six months reached 48% by 2024, showing a significant increase from 37% in 2012. There was global progress in breastfeeding: 70 out of 100 countries reported an increase in exclusive breastfeeding rates since 2017. At the 78th World Health Assembly in 2025, WHO Member States adopted a resolution to extend the Global Nutrition Targets for improving maternal, infant, and young child nutrition from 2025 to 2030. The targets remain vital for identifying priority areas for action and catalyzing global change, aligning with the 2030 Sustainable Development Goals agenda [2,36]. A systematic review published in 2024 shows that, although breastfeeding rates in Europe are unsatisfactory (reaching 25% in 2019), there is a slight upward trend in breastfeeding in Romania in recent years [37,38].

When interpreted in relation to the Baby-Friendly Hospital Initiative (BFHI), our findings suggest that key recommended practices are inconsistently implemented in Romanian maternity settings [39]. Low rates of early breastfeeding initiation, suboptimal skin-to-skin contact, and the frequent use of mixed feeding during hospitalization indicate gaps in adherence to core BFHI principles. Strengthening the implementation of these evidence-based practices could represent a critical step toward improving breastfeeding outcomes at the population level.

Only 69.7% of mothers from our study reported being encouraged by medical staff to initiate breastfeeding during the first contact with their infant, and in 10.6% of cases, initiation of breastfeeding occurred immediately after birth. WHO reports that early initiation of breastfeeding occurs in approximately 46% of newborns being put to breast within one hour of birth, with the goal being 70% by 2030 [2]. Although lactation support during hospitalization did not reach statistical significance in univariate analysis (OR = 0.608, 95% CI: 0.353–1.050, *p* = 0.074), the direction of the association suggests that the absence of support may be linked to a lower likelihood of exclusive breastfeeding. This finding highlights a potential beneficial role of medical staff involvement in supporting breastfeeding outcomes. This finding is consistent with previous evidence showing that structured lactation support interventions can reduce the risk of early breastfeeding discontinuation and improve breastfeeding outcomes [40]. However, previous evidence also suggests that the effectiveness of breastfeeding support interventions may vary across settings and populations, with some studies reporting modest or non-significant effects [41].

The independent association between lactation support during hospitalization and exclusive breastfeeding observed in multivariate analysis suggests that the absence of support may reduce the likelihood of exclusive breastfeeding, underscoring the important role of healthcare professionals in supporting breastfeeding success. These findings are consistent with evidence from systematic reviews and meta-analyses showing that structured breastfeeding support interventions, including those delivered by trained healthcare professionals, improve breastfeeding initiation, exclusivity and duration [41,42]. However, the effectiveness of such interventions may vary depending on the setting and type of support provided, as suggested by broader evidence syntheses [41].

A high percentage of mothers in our study (20.4%) gave birth prematurely, making breastfeeding initiation more difficult in this age group. Breastfeeding difficulties related to infant conditions were reported by 11.8% of participants, including prematurity, ankyloglossia, reflux, and neonatal complications. Investment in Infant and Young Child Feeding (IYCF) in humanitarian settings supports mothers who breastfeed their babies [43]. However, lack of space and privacy, as well as poor sanitation, are critical issues, along with the emotional distress experienced by mothers in emergencies. Therefore, adequate and targeted support is needed for the most vulnerable populations [44,45].

These findings highlight the complex interplay between medical necessity and breastfeeding support in the care of preterm infants. Admission to neonatal units, while often unavoidable, introduces structural barriers such as delayed maternal contact and reduced opportunities for direct breastfeeding. In addition, maternal stress, concerns about infant fragility, and the need for scheduled feeding regimens may further interfere with breastfeeding establishment. Targeted interventions, including kangaroo care and early access to breast pumps, may help mitigate these challenges and improve outcomes in this vulnerable population.

Initiating skin-to-skin contact (SSC) within the first hour after birth stimulates early breastfeeding in the delivery room; however, for medical reasons, prematurity, or maternal or fetal pathology, this first mother–infant contact is delayed. There is solid scientific evidence on the importance of this unique period for both mother and infant, individually and in relation to each other, offering vital benefits for health, regulation and bonding in the short and long term. However, worldwide, clinical practice lags [14,34,46,47]. Although the World Health Organization (WHO) recommends immediate, continuous and uninterrupted skin-to-skin contact after birth, initiating breastfeeding within the first hour of life, newborns are still separated from their mothers during this period in many settings. Our study was conducted in a post-COVID-19 period, and the WHO recommends continuing immediate skin-to-skin contact and early and exclusive breastfeeding during the COVID-19 epidemic, as the benefits substantially outweigh the potential risks of transmission and illness associated with the disease [35,48,49,50]. Maternal medical conditions potentially affecting lactation were reported by 12% of our participants, most commonly thyroid disorders and polycystic ovary syndrome. Mothers may find their thyroid levels change with pregnancy and childbirth, which is why frequent testing of mothers is recommended. Depending on the medication, your baby’s levels may also need to be checked regularly postpartum [51,52].

Interestingly, although mothers delivering in private hospitals reported more favorable early breastfeeding practices, this did not translate into significantly higher rates of exclusive breastfeeding for up to six months. This apparent dissociation suggests that early hospital-based advantages may not be sufficient to sustain breastfeeding in the absence of continued support after discharge. It is possible that postnatal factors, including access to community support, maternal return to work, and family or societal influences, play a more decisive role in long-term breastfeeding outcomes.

The disparities observed between private and public hospital practices reveal deep-seated structural and socioeconomic inequities within the Romanian healthcare system. While private hospital mothers benefited from higher rates of prenatal education and more immediate postnatal support, our findings suggest this is largely a function of personal financial resources rather than a systemic standard. The fact that public hospitals, which care for the majority of the population and a higher proportion of lower-income families, reported significantly longer delays in mother–infant contact and lower staff encouragement is particularly concerning. The differences in staff encouragement (significantly higher in private settings; *p* = 0.013) likely reflect the disparate staffing ratios and workloads between the two sectors. In the public system, understaffed maternity units may prioritize rigid clinical routines over the time-intensive support required for successful breastfeeding initiation. These findings indicate that the public sector requires urgent policy intervention to decentralize lactation support and standardize “Baby-Friendly” protocols, ensuring that a mother’s socioeconomic status does not dictate her infant’s access to the lifelong benefits of early breastfeeding initiation.

A notable finding in this study is that while private hospitals demonstrated superior early clinical practices (e.g., rooming-in and early initiation), these advantages did not result in higher exclusive breastfeeding rates at six months compared to public hospitals. This suggests a “post-discharge support gap.” While the private sector may provide a more supportive “Golden Hour,” the long-term success of breastfeeding is heavily influenced by factors that transcend the birth setting, such as the lack of community-based lactation consultants, insufficient workplace protections, and the widespread availability of breast milk substitutes. In the Romanian context, the initial institutional advantage appears to be neutralized by a broader lack of longitudinal support, highlighting that hospital-based interventions must be bridged with robust community follow-up to sustain exclusive breastfeeding through the first six months.

Only 25.8% of mothers sought the help of a certified lactation consultant, although during hospitalization, a high percentage (77.9%) of mothers reported receiving breastfeeding support from medical staff. The relatively low utilization of certified lactation consultants, partly explained by financial constraints, raises concerns regarding inequitable access to specialized breastfeeding support. Mothers with limited financial resources may be disproportionately affected, potentially widening existing disparities in breastfeeding outcomes. Addressing these barriers through policy measures, such as reimbursement or integration of lactation services into standard postnatal care, could improve access and equity.

The prominent role of family members, social networks, and online communities as sources of breastfeeding information underscores the importance of social and cultural influences in shaping maternal behavior. While these informal networks can provide valuable emotional support, they may also contribute to the dissemination of inconsistent or non-evidence-based advice. This highlights the need for healthcare professionals to engage more actively in antenatal and postnatal education, ensuring that accurate and accessible information reaches mothers across different social contexts.

This study has several limitations that should be considered when interpreting the findings. First, the use of an online, self-administered questionnaire may have introduced selection bias, with a higher likelihood of participation among mothers with higher education levels and access to digital platforms. Second, the reliance on self-reported data may be subject to recall bias and social desirability bias. Third, the cross-sectional design precludes causal inferences between identified factors and breastfeeding outcomes. Fourth, although the sample included participants from both urban and rural areas, the predominance of urban respondents may limit the generalizability of the findings to the broader Romanian population. Finally, the questionnaire was developed specifically for this study based on existing literature and adapted to the Romanian context; face validity was assessed through pre-testing with five mothers with recent breastfeeding experience. However, internal consistency (e.g., Cronbach’s alpha) and construct validity analyses were not performed, as the primary aim of the study was epidemiological assessment rather than instrument validation. The findings of this study have important implications for clinical practice and health policy. Interventions aimed at improving early breastfeeding initiation, promoting rooming-in, and reducing unnecessary formula supplementation should be prioritized within maternity settings. In addition, expanding access to prenatal education and postnatal lactation support may help sustain breastfeeding beyond the immediate postpartum period. A coordinated, multi-level approach that integrates hospital practices with community-based support systems is likely necessary to achieve meaningful improvements in breastfeeding outcomes.

## 5. Conclusions

Our study demonstrates that some progress is being made in protecting and supporting breastfeeding. However, significant challenges remain in supporting and encouraging mothers to optimally feed their infants.

Additional investment and bold policy action are needed to promote and support breastfeeding from the first hour of life, for both term and preterm infants, in all maternity hospitals in Romania when medically indicated. This may be achieved through several measures. First, standardization of “zero-separation” protocols should be implemented. Hospitals must implement protocols that facilitate skin-to-skin contact in the operating room immediately following cesarean sections for stable dyads. Second, rooming-in should be structurally mandated. Hospitals should prioritize architectural and staffing reorganizations to support 24 h rooming-in as the default care model. Third, equitable access to prenatal and postnatal support should be ensured. Given that breastfeeding success and access to lactation consultants are significantly higher among mothers in the private sector with higher incomes, public health policy must integrate International Board Certified Lactation Consultant (IBCLC)-led support into the standard public maternity package. Finally, the BFHI standards should be implemented. The high rate of formula supplementation in our cohort justifies a formal national requirement for hospitals to seek Baby-Friendly Hospital Initiative (BFHI) accreditation, which provides a systematic framework to eliminate unnecessary medical interference and support maternal self-efficacy.

By shifting from a medicalized birth model to a patient-centered support framework, the Romanian healthcare system can move toward achieving the WHO global target of 50% exclusive breastfeeding, ensuring that both term and preterm infants receive the critical protection offered by human milk.

## Figures and Tables

**Table 1:** Maternal and neonatal characteristics and perinatal practices (*n* = 357).

Variable	Group	Number of Respondents (*n* = 357)
Maternal age at study inclusion	Mean ± SD	36.4 years ± 8.15
Maternal age at primiparity	Mean ± SD	28.5 years ± 5.2
Residence	Rural	82 (23%)
	Urban	275 (77%)
Education	Primary/lower education (≤8 years of schooling)	2 (0.6%)
	Vocational training/high school without a diploma	27 (7.6%)
	High school graduate	40 (11.2%)
	University degree (Bachelor’s)	152 (42.6%)
	Postgraduate studies (Master’s/PhD)	136 (38.1%)
Income	Very low (under 4000 RON)	38 (10.6%)
	Low (4000 to 8000 RON *)	131 (36.7%)
	Medium (8001 to 12,000 RON)	99 (27.7%)
	High (over 12,000 RON)	89 (24.9%)
Smoker	Yes	76 (21.3%)
	No	259 (72.5%)
	Occasionally	22 (6.2%)
Number of children	1 child	183 (50.8%)
	2 children	120 (32.2%)
	3 children	36 (12.5%)
	More than 3 children	18 (4.5%)
Attended Prenatal breastfeeding classes	Yes	83 (23.2%)
	No	274 (76.8%)
Type of birth	Vaginal	138 (38.7.%)
	C-section	195 (54.6%)
	C-section with general anesthesia	24 (6.7%)
Reason for C-section	Planned C-section due to medical reasons	112 (50.7%)
	Previous C-section	60 (27.1%)
	Emergency C-section	49 (22.2%)
Gestational age at birth	More than 37 weeks (term)	284 (79.6%)
	Between 34 and 37 weeks	58 (16.2%)
	Between 32 and 34 weeks	8 (2.2%)
	Between 28 and 32 weeks	1 (0.3%)
	Between 24 and 28 weeks	6 (1.7%)
Birth weight	Over 4000 g	20 (5.6%)
	Between 2500 g and 4000 g	292 (81.8%)
	Between 1500 g and 2500 g	38 (10.6%)
	Between 1500 and 1000 g	2 (0.6%)
	Under 1000 g	5 (1.4%)
Interval between delivery and first maternal hold	Immediately	127 (35.6%)
	After 5 min	44 (12.3%)
	After 30 min	14 (3.9%)
	After one hour	28 (7.8%)
	After several hours	133 (37.3%)
	Does not remember	11 (3.1%)
Duration of initial skin-to-skin contact following delivery	Less than 30 min	213 (59.7%)
	More than 30 min	42 (11.8%)
	One hour or more	39 (10.9%)
	Several hours	32 (9%)
	Does not remember	31 (8.7%)
Provider-initiated breastfeeding support at initial holding	Yes	249 (69.7%)
	No	108 (30.3%)
Timing of breastfeeding initiation	Immediately after delivery	38 (10.6%)
	One hour after delivery	73 (20.4%)
	Less than 12 h after delivery	111 (31.1%)
	More than 12 h after delivery	64 (17.9%)
	More than 24 h after delivery	55 (15.4%)
	Never breastfed	16 (4.5%)
In-hospital breastfeeding support	Yes	278 (77.9%)
	No	71 (19.9%)
	Offered but refused	8 (2.2%)
Infant care location	Rooming-in	187 (52.4%)
	Neonatology Ward	170 (47.6%)

Note. * Income was calculated based on medium household incomes in Romania according to public documents available. 12,000 RON is approximately 2355 EUR.

**Table 2:** Maternal and neonatal characteristics and perinatal practices in public hospitals vs. private practice (*n* = 349).

Variable	Group	Public Hospital *n* = 264	Private Practice *n* = 85	*p* (Test)
Residence	Rural	65 (24.6%)	16 (18.8%)	0.27
	Urban	199 (75.4%)	69 (81.2%)	(Chi-square)
Education	Primary/lower education (≤8 years of schooling)	1 (0.4%)	0 (0%)	0.051 (Fisher)
	Vocational training/high school without diploma	22 (8.3%)	5 (5.9%)	
	High school graduate	36 (13.6%)	3 (3.5%)	
	University degree (Bachelor’s)	109 (41.3%)	39(45.9%)	
	Postgraduate studies (Master’s/PhD)	96 (36.4%)	38 (44.7%)	
Income	Very low (under 4000 RON)	34 (12.9%)	2 (2.4%)	**0.001** **(Chi-square)**
	Low (4000 to 8000 RON **)	97 (36.7%)	31 (36.5%)	
	Medium (8001 to 12,000 RON)	79 (29.9%)	20 (23.5%)	
	High (over 12,000 RON)	54(20.5%)	32 (37.6%)	
Smoker	Yes	58 (22.0%)	15 (17.6%)	0.671 (Fisher)
	No	190 (72%)	64 (75.3%)	
	Occasionally	16 (6.1%)	6 (7.1%)	
Number of children	1 child	134 (50.8%)	46 (54.1%)	**0.030** **(Fisher)**
	2 children	85 (32.2%)	32 (37.6%)	
	3 children	33 (12.5%)	2 (2.4%)	
	More than 3 children	12 (4.5%)	5 (5.9%)	
Attended prenatal breastfeeding classes	Yes	49 (18.6%)	31 (36.5%)	**0.001** **(Chi-square**)
	No	215 (81.4%)	54 (63.5%)	
Type of birth	Vaginal	112 (42.4%)	18 (21.2%)	**0.001** **(Chi-square)**
	C-section	133 (50.4%)	62 (72.9%)	
	C-section with general anesthesia	19 (7.2%)	5 (5.9%)	
Reason for C-section	Planned C-section due to medical reasons	77 (29.2%)	35 (41.2%)	**0.004** **(Chi-Square)**
	Previous C-section	42 (15.9%)	18 (21.2%)	
	Emergency C-section	34 (12.9%)	15 (17.6%)	
Gestational age at birth	More than 37 weeks (term)	210 (76.1%)	76 (89.4%)	0.101 (Fisher)
	Between 34 and 37 weeks	49 (18.6%)	8 (9.4%)	
	Between 32 and 34 weeks	7 (2.7%)	1 (1.2%)	
	Between 28 and 32 weeks	1 (0.4%)	0 (0%)	
	Between 24 and 28 weeks	6 (2.3%)	0 (0%)	
Birth weight	Over 4000 g	13 (4.9%)	6 (7.1%)	**0.042** **(Fisher)**
	Between 2500 g and 4000 g	209 (79.2%)	76 (89.4%)	
	Between 1500 g and 2500 g	35 (13.3%)	3 (3.5%)	
	Between 1500 and 1000 g	2 (0.8%)	0 (0%)	
	Under 1000 g	5 (1.9%)	0 (0%)	
Interval between delivery and first maternal hold	Immediately	87 (33.0%)	32 (37.6%)	**0.0001** **(Fisher)**
	After 5 min	36 (13.6%)	8 (9.4%)	
	After 30 min	8 (3.0%)	6 (7.1%)	
	After one hour	11 (4.2%)	17 (20.0%)	
	After several hours	113 (42.8%)	20 (23.5%)	
	Does not remember	9 (3.4%)	2 (2.4%)	
Duration of initial skin-to-skin contact following delivery	Less than 30 min	175 (66.3%)	36 (42.4%)	**0.001** **(Chi-square)**
	More than 30 min	29 (11.0%)	12 (14.1%)	
	One hour or more	20 (7.6%)	16(18.8%)	
	Several hours	14 (5.3%)	16 (18.8%)	
	Does not remember	26 (9.8%)	5 (5.9%)	
Provider-initiated breastfeeding support at initial holding	Yes	177 (67.0%)	69 (81.2%)	**0.013** **(Chi-square)**
	No	87 (33.0%)	16 (18.8%)	
Timing of breastfeeding initiation	Immediately after delivery	21 (8.0%)	11 (12.9%)	**0.001** **(Chi-square)**
	One hour after delivery	38 (14.4%)	34 (40.0%)	
	Less than 12 h after delivery	84 (31.8%)	26 (30.6%)	
	More than 12 h after delivery	59 (22.3%)	5 (5.9%)	
	More than 24 h after delivery	48 (18.2%)	7 (8.2%)	
	Never breastfed	14 (5.3%)	2 (2.4%)	
In-hospital breastfeeding support	Yes	201 (76.1%)	75 (88.2%)	0.059 (Fisher)
	No	56 (21.2%)	9 (10.6%)	
	Offered but refused	7 (2.7%)	1 (1.2%)	
Infant care location	Rooming-in	135 (51.1%)	47 (55.2%)	0.505 (Chi-square)
	Neonatology Ward	129 (48.9%)	38 44.7%)	

Note. Bold was used to highlight statistically significant results. ** Income was calculated based on medium household incomes in Romania according to public documents available. 12,000 RON is approximately 2355 EUR.

**Table 3:** Maternal sociodemographic and perinatal characteristics according to gestational age at delivery (<34 weeks, 34–36 weeks, and ≥37 weeks). Data are presented as *n* (%). *p*-values were calculated using Pearson’s chi-square test or Fisher’s exact test, as appropriate.

Variable	Group	<34 Weeks *n* = 15	34–37 Weeks *n* = 57	>37 Weeks *n* = 277	*p*-Value
Residence	Rural	5 (33.3%)	11 (19.3%)	65 (23.5%)	0.506
	Urban	10 (66.7%)	46 (80.7%)	212 (76.5%)	
Education	Primary/Lower education (≤8 years of schooling)	0 (0%)	0 (0%)	1 (0.4%)	0.407
	Vocational training/high school without a diploma	0 (0%)	4 (7.0%)	23 (8.3%)	
	High school graduate	4 (26.7%)	9 (15.8%)	26 (9.4%)	
	University degree (Bachelor’s)	4 (26.7%)	25 (43.9%)	119 (43.0%)	
	Postgraduate studies (Master’s/PhD)	7 (46.7%)	19 (33.3%)	108 (39.0%)	
Income	Very low (under 4000 RON)	1 (6.7%)	6 (10.5%)	29 (10.5%)	0.872
	Low (4000 to 8000 RON **)	4 (26.7%)	23 (40.4%)	101 (36.5%)	
	Medium (8001 to 12,000 RON)	6 (40.0%)	17 (29.8%)	76 (27.4%)	
	High (over 12,000 RON)	4 (26.7%)	11 (19.3%)	71 (25.6%)	
Smoker	Yes	4 (26.7%)	10 (17.5%)	59 (21.3%)	0.943
	No	10 (66.7%)	43 (75.4%)	201 (72.6%)	
	Occasionally	1 (6.7%)	4 (7.0%)	17 (6.1%)	
Number of children	1 child	9 (60.0%)	27 (47.4%)	144 (52.0%)	0.515
	2 children	6 (40.0%)	19 (33.3%)	92 (33.2%)	
	3 children	0 (0%)	9 (15.8%)	26 (9.4%)	
	More than 3 children	0 (0%)	2 (3.5%)	15 (5.4%)	
Attended prenatal breastfeeding classes	Yes	4 (26.7%)	10 (17.5%)	66 (23.8%)	0.554
	No	11 (73.3%)	47 (82.5%)	211 (76.2%)	
Type of birth	Vaginal	3 (20.0%)	9 (15.8%)	118 (42.6%)	0.002
	C-section	11 (73.3%)	42 (73.7%)	142 (51.3%)	
	C-section with general anesthesia	1 (6.7%)	6 (10.5%)	17 (6.1%)	
Reason for C-section	Planned C-section due to medical reasons	3 (20.0%)	25 (43.9%)	84 (30.3%)	<0.001
	Previous C-section	1 (6.7%)	11 (19.3%)	48 (17.3%)	
	Emergency C-section	8 (53.3%)	12 (21.1%)	29 (10.5%)	
Birth weight	Over 4000 g	0 (0%)	0 (0%)	19 (6.9%)	<0.001
	Between 2500 g and 4000 g	3 (20.0%)	34 (59.6%)	248 (89.5%)	
	Between 1500 g and 2500 g	6 (40.0%)	22 (38.6%)	10 (3.6%)	
	Between 1500 and 1000 g	1 (6.7%)	1 (1.8%)	0 (0%)	
	Under 1000 g	5 (33.3%)	0 (0%)	0 (0%)	
Interval between delivery and first maternal hold	Immediately	2 (13.3%)	12 (21.1%)	105 (37.9%)	<0.001
	After 5 min	0 (0%)	3 (5.3%)	41 (14.8%)	
	After 30 min	0 (0%)	5 (8.8%)	9 (3.2%)	
	After one hour	0 (0%)	0 (0%)	28 (10.1%)	
	After several hours	10 (66.7%)	36 (63.2%)	87 (31.4%)	
	Does not remember	3 (20.0%)	1 (1.8%)	7 (2.5%)	
Duration of initial skin-to-skin contact following delivery	Less than 30 min	12 (80%)	40 (70.2%)	159 (57.4%)	0.18
	More than 30 min	2 (13.3%)	8 (14%)	31 (11.2%)	
	One hour or more	0 (0%)	2 (3.5%)	34 (12.3%)	
	Several hours	0 (0%)	2 (3.5%)	28 (10.1%)	
	Does not remember	1 (6.7%)	5 (8.8%)	25 (9%)	
Provider-initiated breastfeeding support at initial holding	Yes	6 (40%)	40 (70.2%)	200 (72.2%)	0.03
	No	9 (60.0%)	17 (29.8%)	77 (27.8%)	
Timing of breastfeeding initiation	Immediately after delivery	0 (0%)	3 (5.3%)	29 (10.5%)	<0.001
	One hour after delivery	1 (6.7%)	5 (8.8%)	66 (23.8%)	
	Less than 12 h after delivery	1 (6.7%)	13 (22.8%)	96 (34.7%)	
	More than 12 h after delivery	6 (40%)	35(61.4%)	78(28.2%)	
	More than 24 h after delivery	5 (33.3%)	23 (40.4%)	27 (9.7%)	
	Never breastfed	7 (46.7%)	1 (1.8%)	8 (2.9%)	
In-hospital breastfeeding support	Yes	10 (66.7%)	47 (82.5%)	219 (79.1%)	0.61
	No	5 (33.3%)	9 (15.8%)	51 (18.4%)	
	Offered but refused	0 (0.0%)	1 (1.8%)	7 (2.5%)	
Infant care location	Rooming-in	2 (13.3%)	18 (31.6%)	162 (58.5%)	<0.001
	Neonatology Ward	13 (86.7%)	39 (68.4%)	115 (41.5%)	

Note. ** Income was calculated based on medium household incomes in Romania according to public documents available. 12,000 RON is approximately 2355 EUR.

**Table 4:** Univariate logistic regression analysis of factors associated with exclusive breastfeeding.

Variable	OR	95% CI	*p*-Value
Prematurity	0.42	0.24–0.73	0.002
Timing of first holding	1.26	0.83–1.92	0.277
Encouragement at first breastfeeding	1.11	0.70–1.75	0.659
Lactation support during hospitalization	0.61	0.35–1.05	0.074
Rooming-in	3.09	1.99–4.78	<0.001
Mode of delivery	2.73	1.74–4.29	<0.001
Household income	0.85	0.56–1.30	0.442

**Table 5:** Multivariate logistic regression analysis of factors associated with exclusive breastfeeding.

Variable	OR	95% CI	*p*-Value *
Prematurity	0.64	0.35–1.18	0.152
Timing of first holding	0.82	0.49–1.35	0.426
Encouragement at first breastfeeding	1.23	0.72–2.10	0.441
Lactation support during hospitalization	0.63	0.34–1.16	0.136
Rooming-in	2.72	1.71–4.33	<0.001
Mode of delivery	2.44	1.47–4.05	0.001
Household income	0.79	0.50–1.25	0.306

* Model fit: Hosmer–Lemeshow χ^2^ = 3.913, *p* = 0.865; Nagelkerke R^2^ = 0.171; −2 Log likelihood = 435.611.

## Data Availability

The original contributions presented in this study are included in the article. Further inquiries can be directed to the corresponding author.

## Appendix A

Romanian version of the questions included in our questionnaire.

1. Ce vârstă aveți? (ani)
2. Locuiți în mediul: rural/urban
3. Ce studii ați finalizat?
4. Venitul mediu net al familiei dumneavoastră este:
5. Sunteți fumătoare?
6. La ce vârstă ați avut primul copil?
7. Câți copii aveți?
8. Ați urmat cursuri prenatale despre alăptare?
9. De unde ați obținut informații despre alăptare?
10. Ce vârstă are acum cel mai mic dintre copiii dumneavoastră?
11. Unde s-a născut cel mai mic dintre copiii dumneavoastră?
12. Cum s-a născut copilul dumneavoastră?
13. Dacă ați născut prin cezariană, care a fost principalul motiv?
14. La câte săptămâni s-a născut copilul?
15. Cu ce greutate s-a născut copilul?
16. Când ați ținut prima dată copilul în brațe după naștere?
17. Dacă a durat mai mult de 5 min, care a fost motivul?
18. Cât timp ați ținut prima dată copilul în brațe?
19. Ați fost încurajată să puneți copilul la sân prima dată când l-ați ținut în brațe?
20. Când ați pus la sân copilul?
21. Ați primit ajutor de la personalul medical privind alăptarea?
22. Bebelușul a stat cu dumneavoastră în maternitate?
23. Cum a fost hrănit bebelușul în maternitate?
24. Cum ați fost sfătuită să hrăniți bebelușul?
25. Cât timp ați fost sfătuită să țineți copilul la sân?
26. La cât timp după naștere ați observat că aveți lapte matern?
27. Ați oferit suzetă bebelușului?
28. Înainte de naștere, ați plănuit să alăptați?
29. Aveți o afecțiune medicală care ar putea afecta producția de lapte?
30. Dacă da, precizați afecțiunea.
31. Ați urmat vreun tratament medicamentos în perioada alăptării?
32. Dacă da, precizați tratamentul.
33. Bebelușul a avut probleme care au afectat alăptarea?
34. Dacă da, precizați care au fost acestea.
35. Ați fost descurajată de familie sau prieteni în legătură cu alăptarea?
36. Ați simțit presiune din partea personalului medical pentru a renunța la alăptare?
37. Ați apelat la serviciile unui consultant în alăptare?
38. Ați revenit la muncă înainte de 6 luni după naștere?
39. Cum vă hrăniți sau ați hrănit copilul?
40. Alăptați copilul în prezent?
41. Ați reușit sau intenționați să alăptați exclusiv până la 6 luni?
42. Ați întâmpinat dificultăți în alăptare?
43. Dacă da, care au fost acestea?
44. Ce credeți că a contribuit la succesul alăptării?
45. Cum ați descrie experiența alăptării?
46. Cât timp ați alăptat copilul?

## Appendix B

English version of the questionnaire

1. How old are you? (years)
2. Do you live in a rural/urban area?
3. What is the highest level of education you have completed?
4. What is your household’s average monthly net income?
5. Do you smoke?
6. How old were you when you had your first child?
7. How many children do you have?
8. Did you attend prenatal breastfeeding classes?
9. Where did you obtain information about breastfeeding?
10. How old is your youngest child (the one you are thinking about while completing this questionnaire)?
11. Where was your youngest child born?
12. How was your child born?
13. If you delivered by cesarean section, what was the main reason?
14. At how many weeks of gestation was your child born?
15. What was your child’s birth weight?
16. When did you first hold your baby after birth?
17. If it took more than 5 min, what was the reason?
18. How long did you hold your baby the first time?
19. The first time you held your baby, were you encouraged by the maternity staff to put the baby to the breast?
20. When did you first put your baby to the breast?
21. During your hospital stay, did you receive help from medical staff regarding breastfeeding?
22. During your hospital stay, did your baby stay with you?
23. During your hospital stay, how was your baby fed?
24. How were you advised by the maternity staff to feed your baby?
25. How long were you advised to keep your baby at the breast during each feeding?
26. How soon after birth did you first notice that you had breast milk (colostrum or transitional/mature milk)?
27. Did you offer your baby a pacifier?
28. Before giving birth, did you plan to breastfeed?
29. Do you have a medical condition that could affect milk production?
30. If yes, please specify the condition.
31. Did you take any medication while breastfeeding?
32. If yes, please specify the medication.
33. Did your baby have any problems that affected breastfeeding?
34. If yes, please specify these problems.
35. Have family members, friends, or others ever discouraged you from breastfeeding?
36. Did you feel pressured by healthcare professionals to stop breastfeeding?
37. Did you seek help from a lactation consultant?
38. Did you return to work before 6 months postpartum?
39. How do you currently feed, or how did you feed, your baby?
40. Are you currently breastfeeding your child?
41. Did you intend, or were you able, to exclusively breastfeed for 6 months?
42. Did you encounter any difficulties with breastfeeding?
43. If yes, what difficulties did you encounter?
44. What do you believe contributed to your breastfeeding success?
45. How would you describe your breastfeeding experience?
46. How long did you breastfeed your child?
